# The Territorial Medical Officers

**Published:** 1918-04-13

**Authors:** Samuel West

**Affiliations:** President, Royal Medical Benevolent Fund. 11 Chandos Street, Cavendish Square, W. 1.


### THE EDITOR'S LETTER-BOX.

### "The Territorial Medical Officers."

*To the Editor of* The Hospital.

Sir,—Your leader on this subject of March 30 is very timely. It is true that the Territorial medical officers are the worst sufferers, but by no means the only sufferers, among the medical officers who have been on service. Though there is hardship now, there will be greater still when the war is over and the doctors return to civil practice.

To meet these difficulties the Royal Medical Benevolent Fund instituted its War Emergency Fund. Not less than £30,000 will be required, and of this sum nearly £17,000 has been raised from the profession itself, but the appeal must now be made to wider circles. The nation owes a great debt to the medical men who were called out as a profession, and not as individuals. They responded readily, and often at great sacrifice, and it is a debt the country can hardly fail to recognise.

I hope you will lend your influence to make the existence of this War Emergency Fund more widely known, for the necessity of it has been shown by the appeals for help received and the relief already given.—Yours faithfully,

Samuel West,
President, Royal Medical Benevolent Fund.

11 Chandos Street, Cavendish Square, W. 1.
April 5, 1918.

